# Striatal Volume Increases in Active Methamphetamine-Dependent Individuals and Correlation with Cognitive Performance

**DOI:** 10.3390/brainsci2040553

**Published:** 2012-10-30

**Authors:** Reem K. Jan, Joanne C. Lin, Sylvester W. Miles, Rob R. Kydd, Bruce R. Russell

**Affiliations:** 1 School of Pharmacy, Faculty of Medical and Health Sciences, University of Auckland, Private Bag 92019, Auckland 1142, New Zealand; Email: joanne.lin@auckland.ac.nz (J.C.L.); b.russell@auckland.ac.nz (B.R.R.); 2 Centre for Brain Research, Faculty of Medical and Health Sciences, University of Auckland, Private Bag 92019, Auckland 1142, New Zealand; Email: r.kydd@auckland.ac.nz; 3 Waitemata District Health Board, Private Bag 93503, Takapuna, Auckland 0740, New Zealand; Email: wayne.miles@waitematadhb.govt.nz; 4 Department of Psychological Medicine, Faculty of Medical and Health Sciences, University of Auckland, Private Bag 92019, Auckland 1142, New Zealand

**Keywords:** addiction, cognition, drug dependence, Go/No-go, grey matter, magnetic resonance imaging, methamphetamine, putamen, striatum, voxel-based morphometry

## Abstract

The effect of methamphetamine (MA) dependence on the structure of the human brain has not been extensively studied, especially in active users. Previous studies reported cortical deficits and striatal gains in grey matter (GM) volume of abstinent MA abusers compared with control participants. This study aimed to investigate structural GM changes in the brains of 17 active MA-dependent participants compared with 20 control participants aged 18–46 years using voxel-based morphometry and region of interest volumetric analysis of structural magnetic resonance imaging data, and whether these changes might be associated with cognitive performance. Significant volume increases were observed in the right and left putamen and left nucleus accumbens of MA-dependent compared to control participants. The volumetric gain in the right putamen remained significant after Bonferroni correction, and was inversely correlated with the number of errors (standardised z-scores) on the Go/No-go task. MA-dependent participants exhibited cortical GM deficits in the left superior frontal and precentral gyri in comparison to control participants, although these findings did not survive correction for multiple comparisons. In conclusion, consistent with findings from previous studies of abstinent users, active chronic MA-dependent participants showed significant striatal enlargement which was associated with improved performance on the Go/No-go, a cognitive task of response inhibition and impulsivity. Striatal enlargement may reflect the involvement of neurotrophic effects, inflammation or microgliosis. However, since it was associated with improved cognitive function, it is likely to reflect a compensatory response to MA-induced neurotoxicity in the striatum, in order to maintain cognitive function. Follow-up studies are recommended to ascertain whether this effect continues to be present following abstinence. Several factors may have contributed to the lack of more substantial cortical and subcortical GM changes amongst MA-dependent participants, including variability in MA exposure variables and difference in abstinence status from previous studies.

## 1. Introduction

Methamphetamine (MA) is a potent psychostimulant drug that has long lasting and harmful effects on the central nervous system when abused. MA and other amphetamines are abused worldwide and constitute the second most used illicit drugs after cannabis, followed by cocaine and opiates [1]. Abuse of MA is associated with major public health consequences including an increasing number of users requiring treatment (10%, 21% and 20% of all drug-related treatment demand in Europe, the Americas and Oceania, respectively) [1]. Additionally, manufacture of MA through the diversion of precursor substances in clandestine laboratories often involves organised crime and is detrimental to the environment [1].

MA is a potent releaser of the monoamines dopamine (DA), serotonin (5-HT) and noradrenaline (NA) [2]. The effects of MA on noradrenergic transmission are thought to be responsible for the cardiovascular effects experienced following MA intake, such as cardiac arrhythmias and raised blood pressure [3,4], whereas the effects of MA on the dopaminergic system are thought to potentiate its addictive effects [5]. MA has been shown to have toxic effects on DA and 5-HT neurons in animal models of MA addiction, causing both terminal degeneration and cell death [6,7,8,9,10,11]. Post-mortem studies have not reported cell death, yet chronic MA users were shown to have reduced striatal levels of tyrosine hydroxylase and DA transporters (DAT), which are markers of DA nerve terminal integrity and function [12]. However, vesicular monoamine transporter and DOPA decarboxylase levels were unaltered, suggesting long-term striatal neuronal damage that is not permanent [12]. In contrast, a more recent positron emission tomography (PET) study of live recently-abstinent MA abusers found that vesicular DA depletion was increased in comparison to healthy matched control subjects [13]. 

There have been few studies investigating structural differences in the brains of MA users in comparison with healthy controls. Although no differences have been found in total brain volumes of MA abusers [14,15,16,17], a number of structural magnetic resonance imaging (MRI) studies have suggested that MA dependence leads to localised structural changes in both cortical and subcortical brain regions. 

Cortical grey matter (GM) deficits have been reported by most structural MRI studies of MA addiction [16,17,18,19], except one study of abstinent MA abusers which reported increased GM volume in the parietal cortex [20]. Schwartz and colleagues [17] reported GM reductions in the bilateral insula and left middle frontal gyrus of abstinent MA users. The GM deficits in the middle frontal gyrus were thought to be associated with cognitive control deficits [17], whereas deficits in the insula were thought to be related to the behavioural biases associated with drug craving, seeking and addiction [17,21]. Another study compared GM density (GMD) between short-term abstinent (mean abstinence 2.6 ± 1.6 months), long-term abstinent (mean abstinence 30.6 ± 39.2 months) and non-drug using control participants using voxel-based morphometry (VBM) [18]. Short-term abstinent MA abusers had the most extensive decrease in GMD in the right middle frontal gyrus [18]. Long-term abstinent abusers had lower GMD than control participants but were less significantly impaired than short-term abstinent abusers [18]. Therefore, deficits in frontal GM were thought to partially recover following prolonged (six months or longer) abstinence from MA [18]. Nakama and colleagues [19] used region of interest (ROI)-based methods to investigate differences in cortical GM volume between abstinent MA abusers and control participants. They reported decreased cortical GM volumes in six main lobes and 17 cortical sub-regions in MA abusers relative to controls [19]. 

Structural volumetric studies investigating subcortical changes have also been conducted in abstinent MA abusers [14,20]. Larger bilateral volumes of the caudate, nucleus accumbens [20], putamen and globus pallidus were found in MA abusers who had been abstinent for an average of 3–4 months in comparison with healthy control participants [14,20]. MA abusers with larger putamen volumes were found to have a lower lifetime cumulative MA dose and better performance on verbal fluency and speeded motor tasks [14]. It was speculated that striatal structures may undergo an increase in volume as a compensatory response to the neuronal injury caused by chronic MA use, in order to maintain cognitive function [14,20]. 

Regional GM differences have been reported in most structural MRI studies comparing MA abusers with healthy controls. However, most of these studies were conducted in abstinent individuals following variable periods of abstinence. Only one structural brain imaging study of active MA users has previously been performed, although participants were detoxified for 6.64 ± 5.2 days prior to MRI [16]. This study employed cortical and hippocampal pattern matching and found severe GM deficits in MA abusers in the right cingulate, limbic, paralimbic cortices (11.3% below control levels), and hippocampal volumes (7.8% below control levels) [16], but did not investigate volume changes in the striatum. Therefore, the current study aimed to evaluate structural GM changes in active MA-dependent participants in comparison with non-drug using control participants, and whether these changes might be associated with performance on the Go/No-go task, a cognitive task related to impulsivity and response inhibition [22,23]. The Go/No-go task has been used to study dopaminergic modulation of the striatum in nonhuman primates [24,25,26]. Moreover, human studies using functional MRI in conjunction with the Go/No-go task have implicated striatal structures in response inhibition-related neural activity [27,28,29]. Based on prior imaging studies of abstinent users, we hypothesised that active MA-dependent participants would have decreased GMD in the frontal regions and increased GM volume in subcortical structures, specifically the putamen, caudate, globus pallidus and nucleus accumbens. We also hypothesised that increased GM volume in the striatum would correlate with improved performance on the Go/No-go task. 

## 2. Results

### 2.1. Demographics

MA-dependent participants and control participants did not differ significantly in mean age (*t*_(35)_ = −1.72, *p* = 0.095). Chi-square tests were used to test differences in categorical variables; where one or more cells had a count of 5 or less, the Fisher’s Exact Test (FET) statistic was reported. MA-dependent participants and control participants did not differ by gender (FET *p* = 1.000) or alcohol use (*χ*^2^ = 0.23, df = 1, *p* = 0.630). However, tobacco smoking status and cannabis use status differed significantly between groups (FET *p* = 0.000), (Table 1).

**Table 1:** Mean ± SD (range) for demographic characteristics of participants.

	Control Participants (*n* = 20)	MA-Dependent Participants (*n* = 17)
Age (years)	30.9 ± 8.2 (18–46)	35.1 ± 6.6 (22–46)
Gender (males/females)	13/7	12/5
Social drinking (*n*)	10	9
Regular nicotine use	0	14
Cannabis use	0	14
*MA use variables*		
Route of administration (smoking/IV/both)	-	12/3/2
Age at first use (years)	-	23.9 ± 6.6 (12–34)
Duration of use (years)	-	10.2 ± 5.8 (2–25)
Amount of MA used per year (g)	-	119.7 ± 135.5 (12–520)
Lifetime cumulative MA use (g)	-	1442.6 ± 1874.4 (23–5400)

### 2.2. Whole Brain Volumetric Analysis

There were no significant differences in whole brain, GM, white matter or cerebrospinal fluid volumes between groups. 

### 2.3. Subcortical Volumetric Analysis

#### 2.3.1. Between-Group Analysis

**Figure 1:**
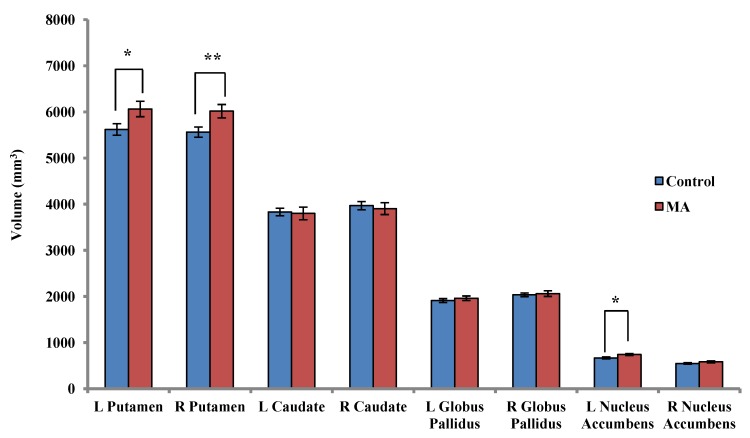
Mean ± standard error subcortical volumes in control participants (*n* = 20) and MA-dependent participants (*n* = 17), * *p* < 0.05 before Bonferroni correction, ** significant after Bonferroni correction.

After controlling for the effect of volumetric scaling factor (VSF), MA-dependent participants had higher volumes than controls in the right putamen (+7.52%; *F*(1,36) = 8.91, *p* = 0.005), left putamen (+7.34%; *F*(1,36) = 5.83, *p* = 0.021) and left nucleus accumbens (+9.85%; *F*(1,36) = 6.63, *p* = 0.015) (Figure 1). However, the only group difference that remained significant after Bonferroni correction was the volume increase in the right putamen of MA-dependent participants. Relative to the control group, MA-dependent participants had greater volumes in the bilateral globus pallidus, and lower volumes in the bilateral caudate, however these results were not statistically significant (Figure 1).

#### 2.3.2. Within-Group Analyses: MA-Dependent Participants

No significant correlations were found between volumes of any of the subcortical structures and duration of MA use or lifetime cumulative MA use. 

**Figure 2:**
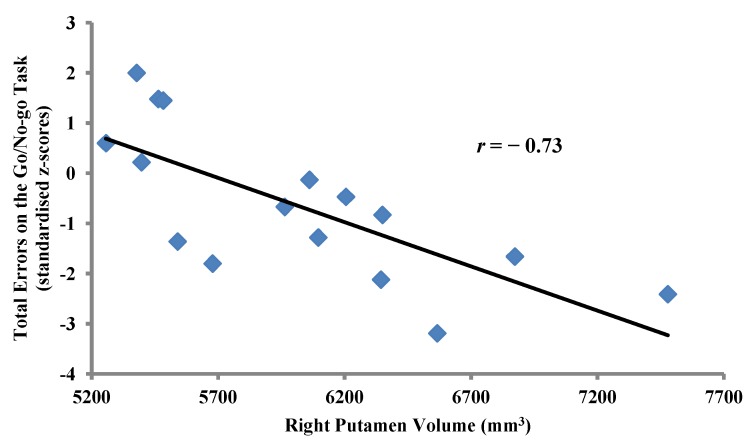
Inverse correlation between right putamen volume and total errors on the Go/No-go task (standardised z-scores) in MA-dependent participants (*n* = 17, Pearson’s *r* = −0.73, *p* = 0.001).

Correlational analysis between the right putamen volume and standardised measures (z-scores) of cognitive performance on the Go/No-go task revealed significant inverse correlations between the right putamen volume and the number of omission errors/false misses (Pearson’s *r* = −0.71, *p* = 0.002) as well as total errors (Pearson’s *r* = −0.73, *p* = 0.001) (Figure 2) on the Go/No-go task.

### 2.4. Voxel-Wise Voxel-Based Morphometry

#### 2.4.1. Between-Group Analysis

**Figure 3:**
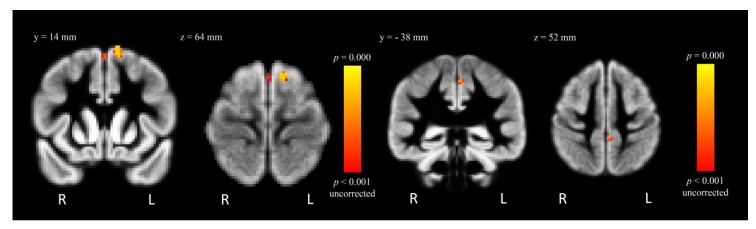
FSL-VBM results from the contrast “Control > MA”, where GMD is decreased for MA-dependent in comparison to control participants within the L SFG (left) and left precentral gyrus (right), *p*_uncorrected_ < 0.001.

**Figure 4:**
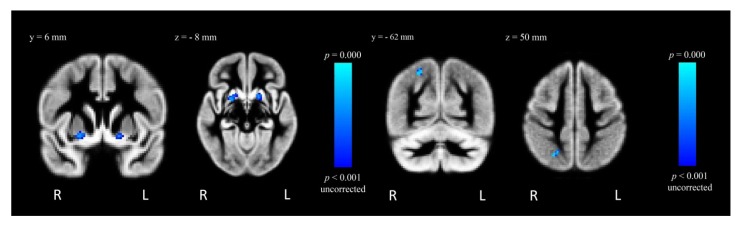
FSL-VBM results from the contrast “MA > Control”, where GMD is greater for MA-dependent in comparison to control participants within the right and left putamen (left) and right superior lateral occipital cortex (right), *p*_uncorrected_ < 0.001.

After controlling for the effect of VSF, MA-dependent participants had decreased GMD in two clusters within the left superior frontal gyrus (L SFG; −23.4% and −26.9%; *t* = 5.25 and *t* = 3.74, respectively, *p*_uncorrected_ = 0.001) relative to control participants (Table 2, Figure 3). The MA-dependent group also showed decreased GMD in a cluster within the left precentral gyrus (−20.3%; *t* = 3.08, *p*_uncorrected_ = 0.001) (Table 2, Figure 3). In contrast, MA-dependent participants had increased GMD within the right putamen (+14.1%; *t* = 3.81, *p*_uncorrected_ = 0.001), the right superior lateral occipital cortex (+24.4%; *t* = 3.37, *p*_uncorrected_ = 0.001) and the left putamen (+18.9%; *t* = 3.74, *p*_uncorrected_ = 0.001) (Table 2, Figure 4). However, these results did not survive correction for multiple comparisons.

**Table 2:** Regions of significance (*p*_uncorrected_ < 0.001) for voxel-wise regressions from FSL-VBM results.

Contrast	Regions	MNI coordinates (mm)			*t*-value	Cluster size (voxels)
		*x*	*y*	*z*		
Control > MA	L SFG	−12	14	66	5.25	48
	L SFG	0	12	60	3.74	14
	L precentral gyrus	−2	−38	52	3.08	13
MA > Control	R putamen	22	2	−10	3.81	55
	R superior lateral occipital cortex	26	−62	50	3.37	42
	L putamen	−16	6	−8	3.74	41

#### 2.4.2. Within-Group Analysis: MA-Dependent Participants

No significant correlations between GMD and lifetime cumulative MA use were found in the MA-dependent group. 

## 3. Discussion

To the best of our knowledge, this is the first structural MRI study of MA-dependent individuals who are active users using VBM and semi-automated subcortical segmentation ROI methods. In accordance with previous volumetric studies of MA abuse [14,15,16,17,30], we found no differences in the whole brain, GM, white matter or cerebrospinal fluid volumes of those with MA dependence in comparison to healthy control participants. 

We report a volume increase within the striatum of individuals with chronic MA dependence who are actively using the drug. Results from the ROI subcortical volumetric analysis showed greater volume in the right putamen of the MA-dependent group, maintaining significance after Bonferroni correction. The MA-dependent group also exhibited enlargement in the left putamen and nucleus accumbens, however these results did not survive Bonferroni correction. Enlargement of the putamen in the MA-dependent group was corroborated by results from the VBM analysis which showed increases in GMD within the bilateral putamen, although these were treated as suggestive rather than absolute due to being uncorrected for multiple comparisons. Enlargement of the putamen [14,20] and nucleus accumbens [20] have been previously reported in those with MA addiction who had been abstinent for an average of 3–4 months. The causative mechanisms for striatal enlargement remain unknown, although neurotrophic effects, inflammatory processes and reactive gliosis have been suggested [14,20]. The hypothesis that neurotrophic effects induce striatal enlargement by increasing neuronal sprouting is limited by findings of a reduction in *N*-acetylaspartate levels in the striatum of those with MA dependence suggesting striatal neuronal loss [31].

The striatum has a high density of dopaminergic terminals and animal studies have shown striatal regions to be most affected by MA-induced DA neurotoxicity [6,9,32,33]. PET studies of the *in vivo* effect of MA on monoaminergic transmission have reported decreased presynaptic DAT and postsynaptic D2 receptor availability in the striatum of people with MA dependence [34,35,36,37,38]. Striatal enlargement has been observed in other conditions which are associated with depletions in DA neurotransmission including cocaine dependence [39,40] and schizophrenia treated with typical anti-dopaminergic neuroleptics [41,42,43]. The neuropathology underlying the striatal enlargement observed in these studies is not fully understood and specific mechanisms by which DA deficiencies may lead to striatal enlargement remain largely unknown. However, a recent study using a combination of VBM and PET has shown correlations between DA D2/D3 receptor distribution and GM volumes in several brain regions including the striatum [44]. Studies in both schizophrenia treated with typical antipsychotics and cocaine dependence have shown neuroleptic-related and cocaine-induced DA depletion and reductions in dopaminergic transmission [41,45,46], which were thought to be mediating factors in the enlargement of striatal structures [39,40,41,42,43]. This theory is further validated by the observation that striatal volumes normalise after patients with schizophrenia are switched to atypical antipsychotics, which have lower affinity for D2 receptors [41,43]. Therefore, it is possible that the putamen enlargement observed in our study occurred as a compensatory response to MA-induced dopaminergic neurotoxicity within the striatum. 

Jernigan and colleagues observed striatal enlargement in MA-dependent participants and speculated the possibility that the effects of MA on brain volumes may be biphasic, whereby enlargement/trophic effects may occur during early stages of dependence and neurodegenerative effects/volume decreases may occur with longer duration of dependence [20]. However, neither this study nor our own study found correlations between MA exposure duration and brain volumes. Nonetheless, this idea is supported by a previous study which reported striatal enlargement in abstinent MA abusers which reversed in individuals with greater lifetime cumulative MA use [14]. This study also reported an association between putamen enlargement and improved performance on neurocognitive tests amongst abstinent MA-dependent participants [14]. Hence, it was suggested that compensatory striatal volume increases in response to MA-induced damage only occurred during early stages of drug dependence, possibly to maintain cognitive function [14]. In the current study, MA-dependent participants with larger right putamen volumes made fewer errors on the Go/No-go task. In particular, MA-dependent participants made fewer omission errors, which may reflect improved selective attention and faster responding, supporting the hypothesis that putamen enlargement may have occurred in response to MA-induced dopaminergic neurotoxicity as a compensatory response to maintain cognitive function. Active drug use in our study may be considered an early phase of drug dependence, and the effect of abstinence on GM volume in our sample of MA-dependent participants remains unknown. 

Using VBM, we found a bilateral GMD gain in the putamen which did not survive correction for multiple comparisons. However, the ROI subcortical volumetric analysis revealed a significant increase in the right putamen volume which survived Bonferroni correction. Although previous studies which measured subcortical volumes in MA dependence reported bilateral gains in striatal volume, these studies were conducted in abstinent users and the results may not be directly comparable with those in active users [14,20]. Alternatively, there may be a real rightward asymmetry in volumetric abnormality in active MA-dependent participants. For example, Thompson and colleagues reported GM deficits in limbic and paralimbic brain regions of MA-dependent participants which were lateralised to the right hemisphere [16]. Other studies conducted in healthy volunteers and patients with negative radiological findings presenting with neurological complaints have reported hemispheric asymmetries in striatal volumes [47]. A pre-existing rightward hemispheric asymmetry cannot be ruled out in our sample of MA-dependent participants and longitudinal follow-up studies are recommended to ascertain this.

The possibility that the enlarged putamen found in this study may reflect other processes including inflammation, microgliosis [48,49,50] and drug-induced increases in striatal perfusion cannot be completely ruled out. The latter is argued against by evidence that MA dependence in humans has been associated with lower cerebral blood flow [51], including to the anterior cingulate cortex (ACC) [52], insula and putamen [53]. Moreover, DA released by MA has been shown to constrict vessels and reduce blood flow in the rat caudate and putamen [54].

Although not statistically significant, the trend towards volumetric enlargement of the globus pallidus has been previously reported in those abstinent from MA [14,20]. However, the trend towards lower caudate volumes in MA-dependent participants is contrary to previously reported volume increases in the caudate [20], especially given that the caudate has been reported to be more adversely affected by MA addiction than the putamen. For example, in a post-mortem analysis, greater depletion of DA content has been found in the caudate than the putamen of people with chronic MA addiction [55]. Nonetheless, absence of volumetric increases within the caudate of MA-dependent individuals has been previously reported by Chang and colleagues who found an enlarged putamen and globus pallidus in their group of abstinent MA abusers, but not in the caudate [14]. Moreover, active cocaine users were reported to have larger volumetric increases in the putamen than the caudate (9.2% *vs.* 3.4%, respectively) [40]. 

This study found decreased frontal cortical GMD in the LSFG and left precentral gyrus and increased GMD in the right superior lateral occipital cortex of active MA-dependent in comparison to control participants; however, these results did not survive correction for multiple comparisons. Reductions in GMD within the LSFG and left precentral gyrus may be functionally significant. For example, Schwartz and colleagues reported an association between GMD reductions in the LSFG and increased impulsivity, as measured by the delay discounting task in abstinent MA-dependent individuals [17]. Moreover, in a functional MRI study using the Stroop colour-word task, recently abstinent MA-dependent subjects exhibited lower activation of the precentral gyrus in response to the incongruent (conflict) condition compared to control subjects [56]. 

The discrepancy between our study and previous studies in the lack of more substantial cortical and subcortical GM changes may be due to the difference in MA use status of our group in contrast to study participants in other research *i.e.*, active *vs.* abstinent users. It is possible that some volumetric changes may occur during the active use of MA, whilst others may only become apparent following drug abstinence. There has only been one other structural study by Thompson and colleagues in active MA-dependent individuals that reported GM deficits in limbic and paralimbic regions in comparison to controls, and we did not replicate this finding [16]. Although the MA-dependent group in Thompson’s study was reported to have used MA on most of the 30 days prior to entering the study, they were also abstinent from MA for 6.64 ± 5.2 days prior to the scan day [16]. Therefore this group of MA-dependent individuals may not be directly comparable with our sample of active users, whose current active use status was confirmed by a qualitative urine drug test on the scan day. Furthermore, different data acquisition and analytical techniques may have affected the results. For example, Thompson and colleagues employed surface-based cortical GM mapping techniques with high resolution anatomical scans using a 3 T MRI system [16]. In comparison, our images were obtained at 1.5 T and analysed using semi-automated methods including VBM, SIENAX and FIRST within FSL (see Experimental Procedure for details). Lastly, the study by Thompson and colleagues did not examine striatal volumes [16]. Therefore, to the best of our knowledge, the current study is the first structural MRI study to report striatal changes in active MA-dependent individuals.

There are several limitations to the current study. Firstly, the sample size was modest (17 MA-dependent participants, 20 control participants). Secondly, tobacco smoking and cannabis use were significantly more prevalent amongst MA-dependent than control participants. The majority of MA users in New Zealand also use cannabis and smoke tobacco. For example, a New Zealand epidemiological study found that within a six month period during 2010, 87% of frequent MA users also used cannabis and 86% used tobacco [57]. Tobacco smoking has been associated with decreased cortical GMD in the bilateral prefrontal cortex and left dorsal ACC [58]. The effects of tobacco smoking and cannabis use could not be completely separated from those of MA in the statistical analysis and may confound the results. However, studies on the effects of cannabis use on brain structure are sparse and the results are inconsistent. Most studies suggest that cannabis use is not associated with structural brain changes [59,60]. For example, a recent study of amphetamine-type stimulant users using VBM and tract based spatial statistics showed no association between GM volume and cannabis-related parameters [60]. Although a recent VBM study reported larger GM volume in the anterior cerebellum of heavy cannabis users in comparison to controls [61], GM volumes in other regions such as the orbitofrontal cortex, ACC, striatum, amygdala and hippocampus did not significantly differ between groups. Within the heavy cannabis user group, the amount of cannabis use was inversely correlated with GM volume in the amygdala and hippocampus, and the authors suggested that associations between heavy cannabis use and brain structure are complex [61]. This study suffered from several limitations, whereby significant group differences in several potential confounding variables were present. For example, the cannabis users in this study were also nicotine dependent and scored significantly higher than the control group for depression and attention deficit hyperactivity disorder symptoms [61]. Thirdly, our VBM results failed to show statistically significant group differences following correction for multiple comparisons. There may be several reasons for this including the modest sample size and the range of MA exposure variables. For example, the average duration of MA use was 10.2 years but it ranged from 2 to 25 years. Similarly, the average lifetime cumulative MA dose was 1442.6 g with a wide range of 23–5400 g. It is possible that this caused a wash out of effects whereby group differences in GMD may have been diluted. To further investigate this, an exploratory within-group analysis was carried out to assess the relationship between GMD and lifetime cumulative MA dose, however no significant correlations were observed. The lack of a relationship between MA exposure variables and GM volumes may reflect the relative inaccuracy of self-report drug use data, or may suggest that a threshold effect exists for MA-induced neurotoxicity and resulting changes in regional brain volumes [19]. Nonetheless, failure to observe a relationship between amount and recency of drug use and GM volumes has been previously reported in structural MRI studies of stimulant dependence [40,62]. Finally, because this study was cross-sectional, it is impossible to ascertain the causality of GM volume changes and the possibility that putamen enlargement predated MA dependence cannot be ruled out. Prospective studies of human subjects at-risk of developing MA dependence and who have not initiated use would be extremely useful, albeit challenging. Alternatively, longitudinal studies of actively using MA-dependent individuals followed into abstinence would be feasible. 

## 4. Experimental Procedure

### 4.1. Participants

Seventeen adult participants with a history of MA dependence and still actively using MA (five female; age 35.1 ± 6.6 years) were recruited from Community Alcohol and Drug Services in Point Chevalier, Auckland, New Zealand and 20 matched control participants with no previous history of drug addiction (seven female; age 30.9 ± 8.2 years) were recruited by word of mouth and advertisements (Table 1). MA-dependent participants were screened and diagnosed by a consultant psychiatrist using a structured clinical interview (SCID-I, Clinical Trials Version) [63]. Data collected also included detailed questions regarding age at first use, route of administration, average amount of MA use per day, number of days used per week and duration of regular use as well as self-rated level of use (light, regular, heavy) (Table 1). This was used to estimate the lifetime cumulative amount of MA used (Table 1). MA-dependent participants also underwent physical examinations, and blood and urine testing to ensure health. They fulfilled the following inclusion criteria: (1) age between 18 and 45 years and of any ethnicity; (2) diagnosis of MA dependence according to DSM-IV criteria; current MA use was confirmed by qualitative urine drug tests (cut-off 300 μg/L); (3) negative urine toxicology screen (except for MA, cannabis, nicotine and caffeine) and no current or past history of other drug dependence, such as alcohol, cannabis, cocaine, opioids or benzodiazepines; (4) taking no other prescribed medications, except for oral contraceptives and mild analgesics when required.

Control participants fulfilled the same inclusion/exclusion criteria as those with MA dependence but were excluded if they had a history of drug use including MA. Exclusion criteria were: (1) past or present Axis I psychiatric diagnosis (other than MA dependence, but including schizophrenia and major depression); (2) neurological, thyroid, renal, gastrointestinal or cardiovascular disease; (3) clinically significant hepatic disease; (4) past or present illnesses known to affect cognition (e.g., stroke, traumatic brain injury, epilepsy, Parkinson’s disease, neurodegenerative disorders); (5) risk of suicide or violent behaviour; (6) glaucoma; (7) Tourette’s disorder or tics; (8) in females, current pregnancy or lactation; (9) any contraindications for MRI (e.g., claustrophobia and implanted ferromagnetic objects). All procedures were approved by the Northern X Regional Ethics Committee of New Zealand and participants gave written informed consent prior to taking part in this study.

### 4.2. Magnetic Resonance Image Acquisition

All participants underwent structural MRI using a 1.5 T Siemens Magnetom Avanto scanner (Siemens Medical Solutions, Erlangen, Germany). A high resolution T_1_-weighted anatomical magnetically prepared rapid acquisition gradient echo (MPRAGE) sequence was used (144 slices 1.25 mm thick, TR = 2400 ms, TE = 3.61 ms, TI = 1000 ms, flip angle = 8°, FOV = 180 × 240 mm, imaging matrix = 180 × 240), yielding 1.25 mm^3^ isotropic voxel resolution. Two T_1_-weighted MPRAGE sequences were acquired for each participant during the same scanning session. 

### 4.3. Neuropsychological Testing

An automated battery of neuropsychological tests, IntegNeuro (The Brain Resource Company, NSW, Australia), was administered to MA-dependent participants prior to MRI scanning, on the same day. Testing took approximately one hour to complete and was run on a touchscreen platform with task instructions delivered through a headset and verbal responses recorded via a microphone. The IntegNeuro test battery consisted of 13 tests spanning six core general cognition domains. Behavioural measures from the neuropsychological tests were available as “standardised scores” which were normalised for age, gender and years of education and presented as “z-scores”. Standardised norms were pre-established in 1000 healthy participants (data are part of the Brain Resource International Database) [64]. Performance on the Go/No-go task was correlated with volumes which showed group differences following correction for multiple comparisons. Control subjects did not undergo neuropsychological testing; hence z-scores, which take into account standardised norms, were used instead of raw scores for the correlation analysis with significant volumes. The Go/No-go task has been widely used in cognitive psychology research, including drug addiction studies, as a measure of impulsivity and inhibitory control [22,23]. The task had two conditions: Go (“PRESS” written in green), where the subject was asked to respond using the index finger of each hand, and No-go (“PRESS” written in red), where the subject was instructed to make no response. Stimuli were presented for 500 ms and had an inter-stimulus interval of 1 s. Speed and accuracy of response were equally stressed in the instructions. The task was approximately five minutes long and consisted of 28 sequences where the word “PRESS” was presented in the same colour six times in a row. Seven of these sequences were presented in red in a pseudo-random order. Four measures were recorded for each subject, including reaction time, number of false alarms/commission errors (responses to red “PRESS” stimuli which indicate failure to inhibit an anticipated response), number of false misses/omission errors (non-responses to green “PRESS” stimuli which indicate inattention or slow responding) and total errors (sum of commission and omission errors). One subject was excluded from the correlational analysis due to incompletion of neuropsychological testing.

### 4.4. Analysis

Structural data analysis was carried out with Functional Magnetic Resonance Imaging of the Brain (FMRIB) Software Library (FSL, v4.1.8) tools [65]. Images were compiled into standard NIfTI-1 (Neuroimaging Informatics Technology Initiative) format and reoriented to a right-to-left, posterior-to-anterior, inferior-to-superior co-ordinate system. For each participant, one of the acquired T_1_-weighted structural images was linearly registered to the second image using the FMRIB’s Linear Image Registration Tool (FLIRT, v5.5) [66,67]. The two images were then combined, intensity normalised and averaged to produce one average structural image per participant. This process aimed to achieve accurate tissue class probability assignments; the resulting average structural images were used for volumetric analyses. Anatomical labeling was carried out using the Harvard-Oxford cortical and subcortical structural atlases [68].

#### 4.4.1. Whole Brain Volumetric Analysis

Total brain tissue volume, normalised for subject head size, was estimated with Structural Image Evaluation, using Normalisation, of Atrophy (SIENAX), v2.6 [69], part of FSL, v4.1.8 [65]. Brain and skull images were extracted from the single anatomical images [70], then linearly registered to the Montreal Neurological Institute (MNI) 152 subject atlas space [66,67], to obtain a volumetric scaling factor (VSF), used as a normalisation for head size. Next, tissue-type segmentation with partial volume estimation was carried out [71] to calculate total volume of brain tissue, including separate estimates of volumes of GM, peripheral (cortical) GM, white matter and ventricular cerebrospinal fluid. Between-group comparisons in whole brain volumetric measures were performed in SPSS v19 using one-way univariate analysis of variance (ANOVA) analyses, controlling for VSF.

#### 4.4.2. Subcortical Volumetric Analysis

Volumetric analysis of subcortical structures was carried out using FMRIB’s Integrated Registration and Segmentation Tool (FIRST, v1.2) [72]. FIRST is a model-based segmentation/registration tool which uses shape/appearance models constructed from manually segmented images provided by the Center for Morphometric Analysis, Massachusetts General Hospital, Boston. All structural images underwent a two-stage linear registration in order to achieve an accurate and robust pre-alignment of subcortical structures. The first stage was a linear registration of the whole head in each structural image to the non-linear MNI152 template with a 1 × 1 × 1 mm resolution [66]. A subcortical mask which excluded regions outside of the subcortical structures, along with the first stage linear registration alignments were used in the second stage to achieve robust and accurate registration of subcortical structures to the MNI152 template. Following this, the images were transformed back to the native space using the inverse transformation, to allow the subsequent segmentation steps to be performed in the native space with the original voxel intensities. Segmentation of subcortical structures was performed and volumetric masks that represented the structure segmentation were created and a FMRIB’s Automated Segmentation Tool (FAST, v4.1)-based boundary correction method was applied [71], with exceptions including the putamen and globus pallidus where a slightly different surface model was fit. Since MA users were previously reported to have larger volumes of the putamen, globus pallidus [14,20], caudate and nucleus accumbens [20], the subcortical volumetric analysis was confined to these four structures bilaterally. Hence, one-way univariate ANOVA analyses were conducted in SPSS v19, controlling for VSF, to investigate the main effect of group on subcortical volumes. Bonferroni correction was applied and both corrected and uncorrected significant results were reported. Within the MA-dependent group, two correlational analyses were conducted. The first analysis was performed to examine the association between subcortical volume and MA exposure variables such as duration of MA use and lifetime cumulative MA use. The second analysis investigated the relationship between brain volumes that showed significant group differences after Bonferroni correction and cognitive performance on the Go/No-go task. 

#### 4.4.3. Voxel-Wise Voxel-Based Morphometry Analysis

The FSL-VBM tool, v1.1, was used to carry out a VBM style analysis [73,74]. First, structural images were brain-extracted using the Brain Extraction Tool (BET, v2.1) [70]. Tissue-type segmentation was carried out using FAST [71] and produced GM partial volume images, which were then aligned to the MNI152 subject atlas space using the affine registration tool, FLIRT [66,67], followed by non-linear registration using FMRIB’s Non-linear Image Registration Tool (FNIRT, v1.0) [75,76]. The resulting images were averaged to create a study-specific template, to which each participant’s GM image was then non-linearly re-registered. The registered GM partial volume images were then modulated by dividing each voxel of each registered GM image by the Jacobian of the warp field, in order to correct for local expansion or contraction which may have occurred due to the non-linear component of the transformation. The modulated segmented images were then smoothed with an isotropic Gaussian kernel with a full-width at half-maximum of 3 mm. In order to examine the relationship of GMD to group membership and clinical variables, a voxel-wise general linear model was applied using permutation-based non-parametric testing [77], as implemented by the Randomise v2.1 toolbox within FSL. Non-parametric two-sample unpaired *t*-tests were used to analyse regional GMD after controlling for the effect of VSF. Within the MA-dependent group, a separate voxel-wise regression was performed to examine the association between GMD and lifetime cumulative MA use. 

## 5. Conclusions

To the best of our knowledge this is the first study to report striatal enlargement associated with improved cognitive function in MA-dependent individuals who are active users. Future studies comparing structural GM changes and cognitive function in active versus short- and long-term abstinent MA users with matched controls should shed more light on the progression of these changes and the extent of reversibility, if any, following abstinence. 
